# Supplementary material for: High-throughput behavioral screen in C. elegans reveals Parkinson’s disease drug candidates
Source: Commun Biol. 2021 Feb 15;4:203. doi: 10.1038/s42003-021-01731-z (PMC7884385; doi:10.1038/s42003-021-01731-z)
Supplement: Supplementary file 2 — Supplementary Information [file 42003_2021_1731_MOESM2_ESM.pdf]

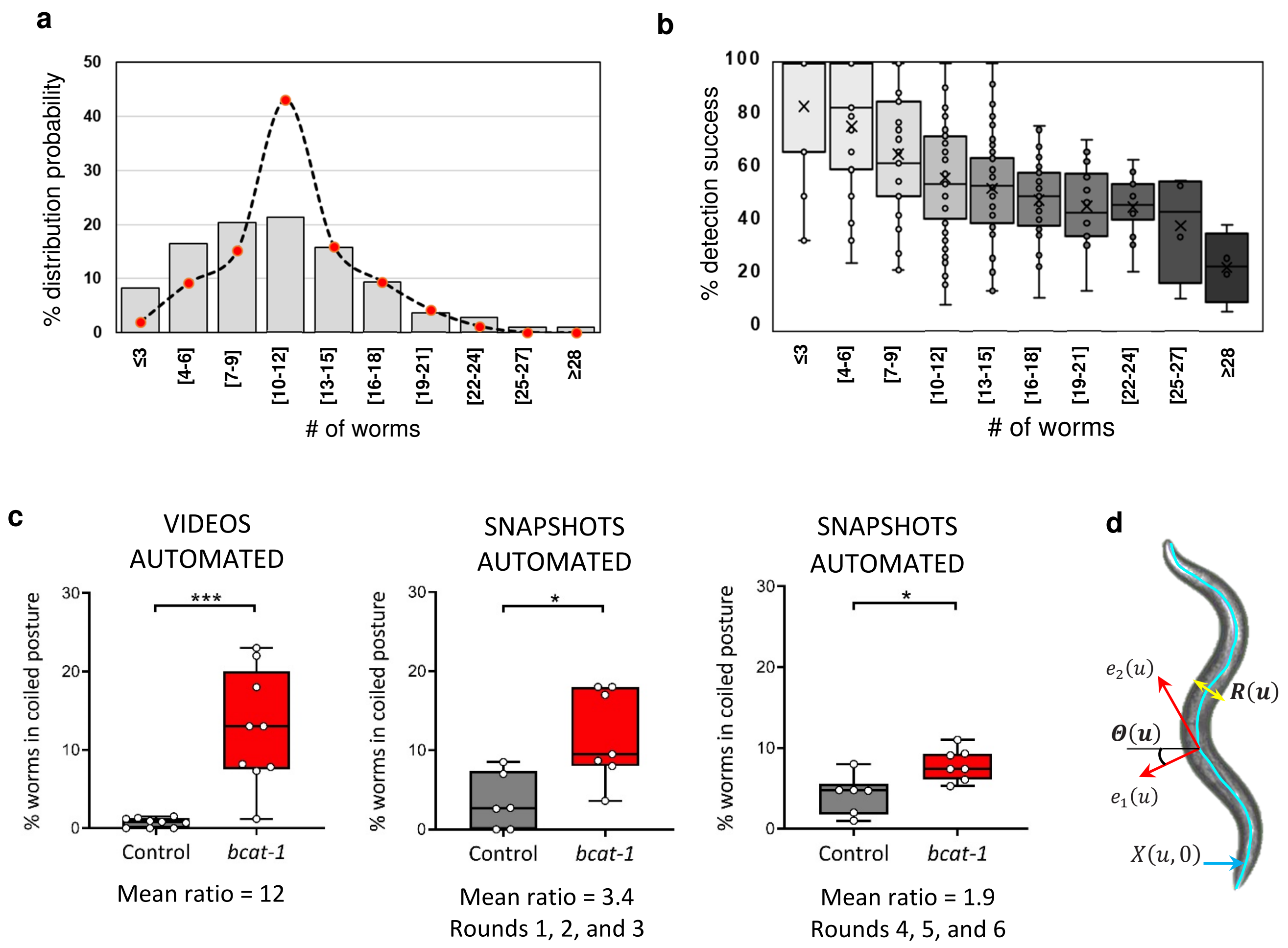

Supplementary Figure 1. (a) Distribution of # animals across 436 wells from 64 tested conditions (bars). Normalized distribution of animals across wells corresponds to the efficacy of worm loading setup (dashed line). The total # of worms tested for each condition is normalized to $n \times 11$ worms distributed across n wells. (b) % detection success decreases with increasing # worms per well, due to greater likelihood of animals overlapping and therefore being censored. n=436 wells totaling 4660 worms. (c) Re-analyzing videos-snapshot comparison test from Figure 2c using only Coiled postures. The Snapshots Automated panels includes curling data for rounds 1, 2, and 3 and rounds 4, 5, and 6, respectively. All rounds of snapshots are captured within a 60min window after transfer to liquid. n = 5 wells totaling 50 worms for control videos, 7 wells totaling 73 worms for *bcat-1* videos, 6 wells totaling 71 worms for control snapshots, 6 wells totaling 41 worms for *bcat-1* snapshots. (d) Illustration of geometric model for *C. elegans* body. Two-tailed *t*-tests. *p<0.05, ***p<0.001. Box plot show minimum, 25th percentile, median, 75th percentile, maximum. Mean of *bcat-1(RNAi)* divided by mean of control RNAi is abbreviated as mean ratio.

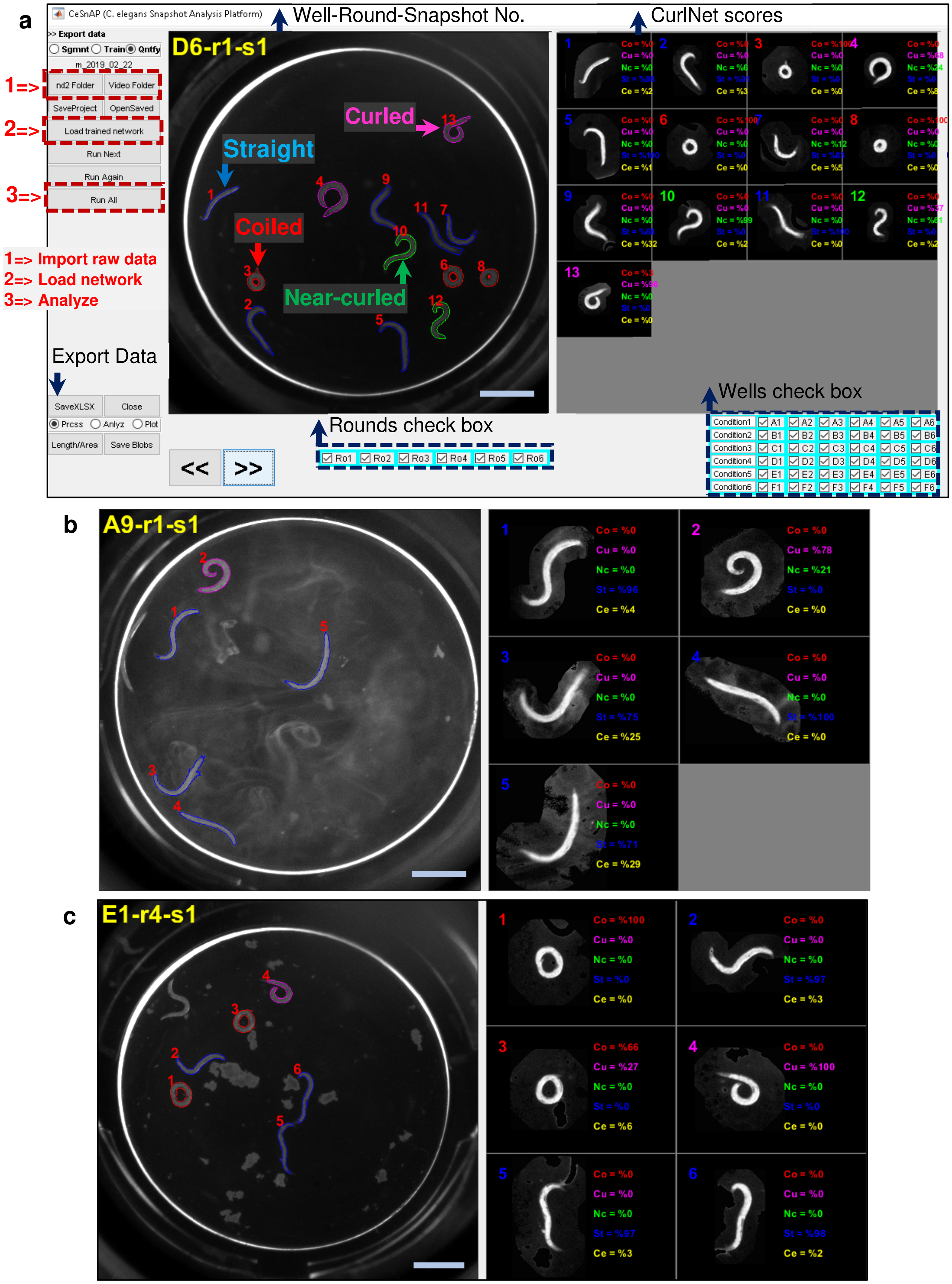
a
CeSnAP (C. elegans Snapshot Analysis Platform)
Well-Round-Snapshot No.
CurlNet scores
>> Export data
Sgmnt Train Qntfy
m_2019_02_22
1=>
nd2 Folder
Video Folder
SaveProject
OpenSaved
2=>
Load trained network
Run Next
Run Again
3=>
Run All
1=> Import raw data
2=> Load network
3=> Analyze
Export Data
SaveXLSX
Close
Prcss Anlyz Plot
Length/Area
Save Blobs
D6-r1-s1
Curled
Straight
Coiled
Near-curled
<<
>>
Rounds check box
Ro1 Ro2 Ro3 Ro4 Ro5 Ro6
Wells check box
Condition1 A1 A2 A3 A4 A5 A6
Condition2 B1 B2 B3 B4 B5 B6
Condition3 C1 C2 C3 C4 C5 C6
Condition4 D1 D2 D3 D4 D5 D6
Condition5 E1 E2 E3 E4 E5 E6
Condition6 F1 F2 F3 F4 F5 F6
b
A9-r1-s1
1 Co = %0 Cu = %0 Nc = %0 St = %96 Ce = %4
2 Co = %0 Cu = %78 Nc = %21 St = %0 Ce = %0
3 Co = %0 Cu = %0 Nc = %0 St = %75 Ce = %25
4 Co = %0 Cu = %0 Nc = %0 St = %100 Ce = %0
5 Co = %0 Cu = %0 Nc = %0 St = %71 Ce = %29
c
E1-r4-s1
1 Co = %100 Cu = %0 Nc = %0 St = %0 Ce = %0
2 Co = %0 Cu = %0 Nc = %0 St = %97 Ce = %3
3 Co = %66 Cu = %27 Nc = %0 St = %0 Ce = %6
4 Co = %0 Cu = %100 Nc = %0 St = %0 Ce = %0
5 Co = %0 Cu = %0 Nc = %0 St = %97 Ce = %3
6 Co = %0 Cu = %0 Nc = %0 St = %98 Ce = %2

Supplementary Figure 2. (a) New analyses in SnapMachine can be simply initiated after loading raw data and a trained neural network. CurlNet performance in identifying target classes of worm postures in front of a noisy background (b) and in wells with debris and bacterial chunks (c). Scale bar, 1mm.

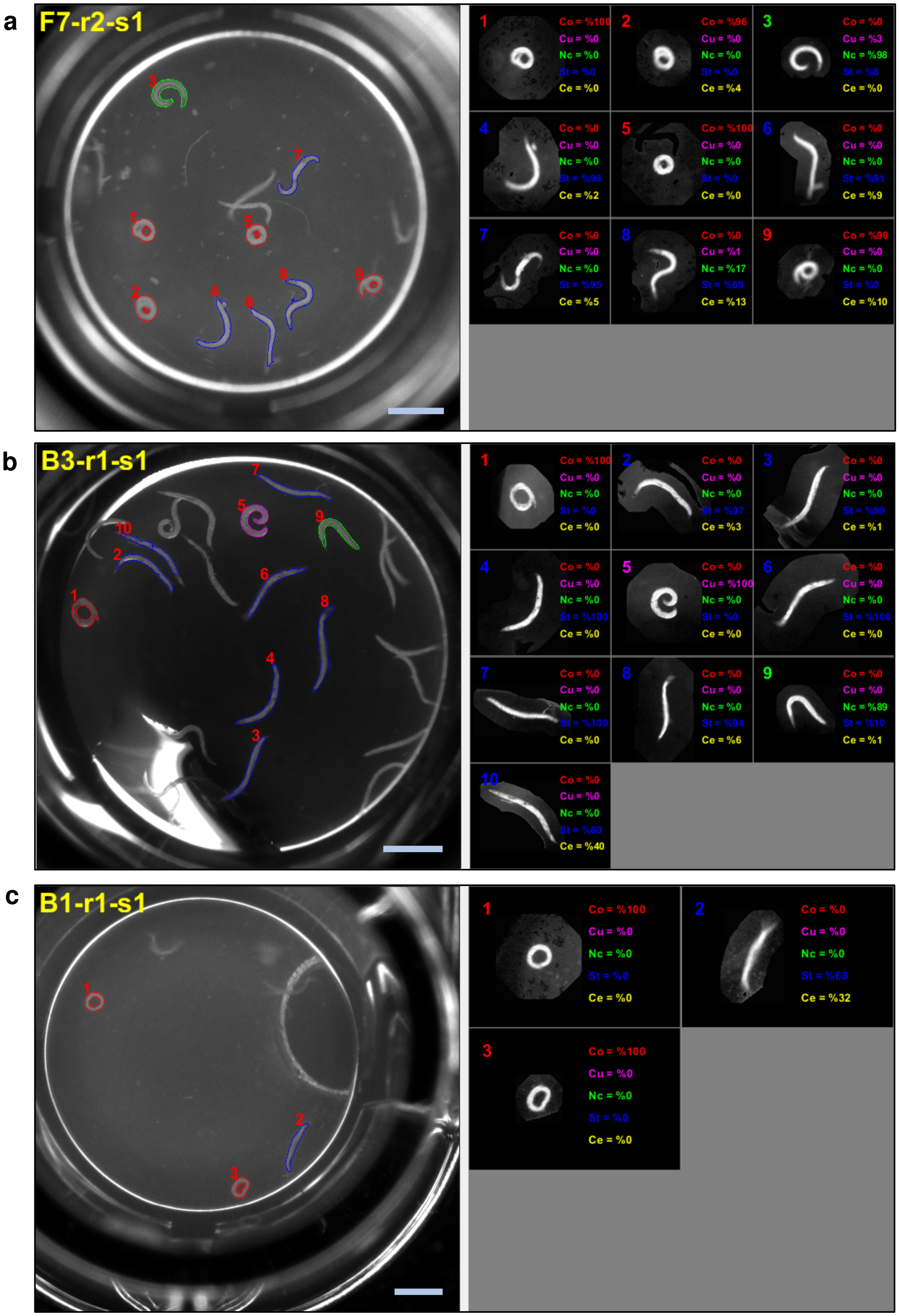
a
F7-r2-s1
1
Co = %100
Cu = %0
Nc = %0
St = %0
Ce = %0
2
Co = %96
Cu = %0
Nc = %0
St = %0
Ce = %4
3
Co = %0
Cu = %3
Nc = %98
St = %0
Ce = %0
4
Co = %0
Cu = %0
Nc = %0
St = %98
Ce = %2
5
Co = %100
Cu = %0
Nc = %0
St = %0
Ce = %0
6
Co = %0
Cu = %0
Nc = %0
St = %91
Ce = %9
7
Co = %0
Cu = %0
Nc = %0
St = %95
Ce = %5
8
Co = %0
Cu = %1
Nc = %17
St = %69
Ce = %13
9
Co = %90
Cu = %0
Nc = %0
St = %0
Ce = %10
b
B3-r1-s1
1
Co = %100
Cu = %0
Nc = %0
St = %0
Ce = %0
2
Co = %0
Cu = %0
Nc = %0
St = %97
Ce = %3
3
Co = %0
Cu = %0
Nc = %0
St = %99
Ce = %1
4
Co = %0
Cu = %0
Nc = %0
St = %100
Ce = %0
5
Co = %0
Cu = %100
Nc = %0
St = %0
Ce = %0
6
Co = %0
Cu = %0
Nc = %0
St = %100
Ce = %0
7
Co = %0
Cu = %0
Nc = %0
St = %100
Ce = %0
8
Co = %0
Cu = %0
Nc = %0
St = %94
Ce = %6
9
Co = %0
Cu = %0
Nc = %89
St = %10
Ce = %1
10
Co = %0
Cu = %0
Nc = %0
St = %60
Ce = %40
c
B1-r1-s1
1
Co = %100
Cu = %0
Nc = %0
St = %0
Ce = %0
2
Co = %0
Cu = %0
Nc = %0
St = %68
Ce = %32
3
Co = %100
Cu = %0
Nc = %0
St = %0
Ce = %0

Supplementary Figure 3. CurlNet performance in identifying target classes of worm postures (a) in an out-of-focus photograph of a well, (b) in an image distorted due to non-uniform meniscus, and (c) in the presence of a bubble and off-centered well. Scale bar, 1mm.

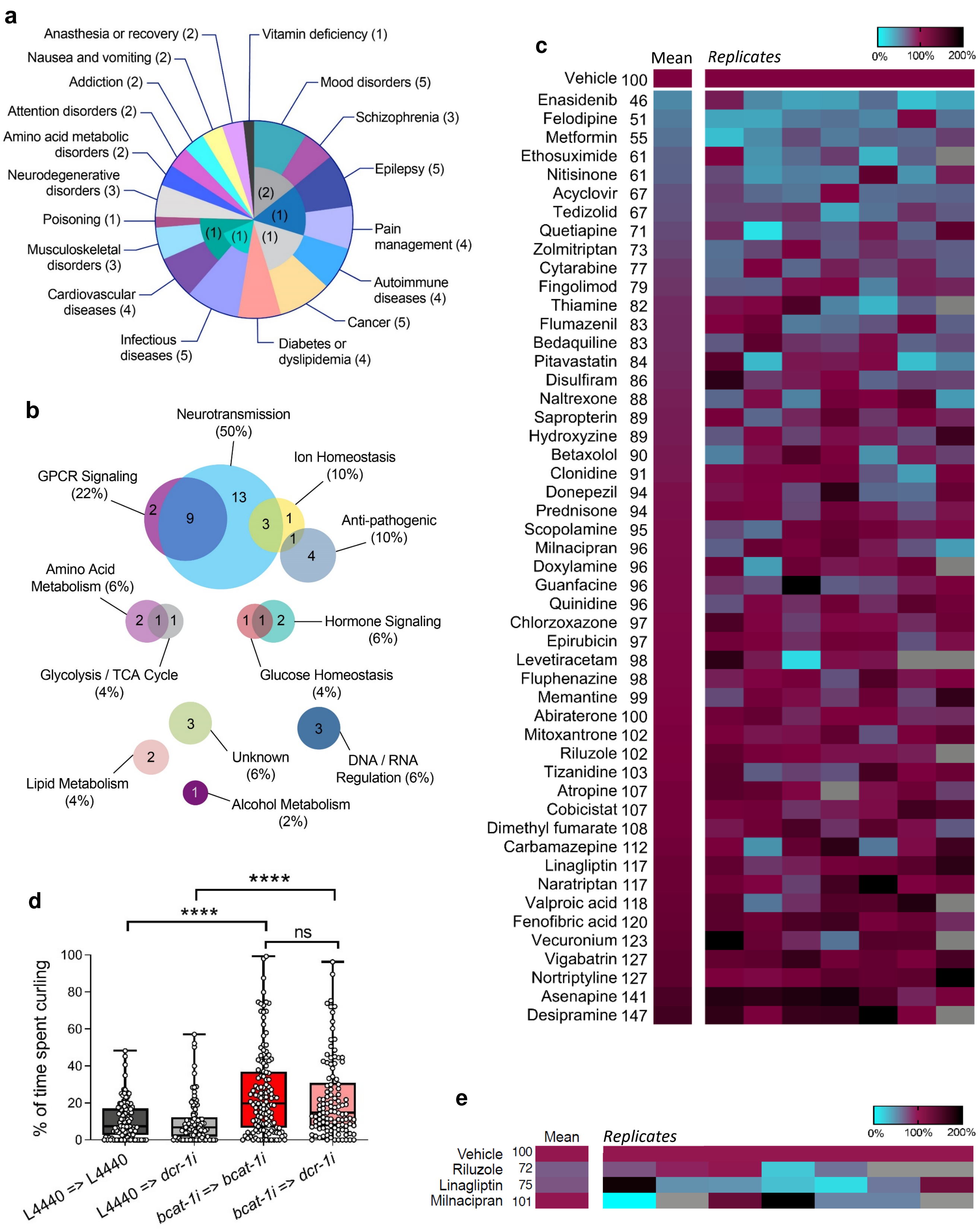
a
Anasthesia or recovery (2)
Nausea and vomiting (2)
Addiction (2)
Attention disorders (2)
Amino acid metabolic disorders (2)
Neurodegenerative disorders (3)
Poisoning (1)
Musculoskeletal disorders (3)
Cardiovascular diseases (4)
Infectious diseases (5)
Diabetes or dyslipidemia (4)
Cancer (5)
Autoimmune diseases (4)
Pain management (4)
Epilepsy (5)
Schizophrenia (3)
Mood disorders (5)
Vitamin deficiency (1)
(2)
(1)
(1)
(1)
(1)
b
Neurotransmission (50%)
GPCR Signaling (22%)
Ion Homeostasis (10%)
Anti-pathogenic (10%)
13
2
9
3
1
1
4
Amino Acid Metabolism (6%)
Glycolysis / TCA Cycle (4%)
Hormone Signaling (6%)
Glucose Homeostasis (4%)
Unknown (6%)
DNA / RNA Regulation (6%)
Lipid Metabolism (4%)
Alcohol Metabolism (2%)
c
Mean
Replicates
0%
100%
200%
Vehicle 100
Enasidenib 46
Felodipine 51
Metformin 55
Ethosuximide 61
Nitisinone 61
Acyclovir 67
Tedizolid 67
Quetiapine 71
Zolmitriptan 73
Cytarabine 77
Fingolimod 79
Thiamine 82
Flumazenil 83
Bedaquiline 83
Pitavastatin 84
Disulfiram 86
Naltrexone 88
Sapropterin 89
Hydroxyzine 89
Betaxolol 90
Clonidine 91
Donepezil 94
Prednisone 94
Scopolamine 95
Milnacipran 96
Doxylamine 96
Guanfacine 96
Quinidine 96
Chlorzoxazone 97
Epirubicin 97
Levetiracetam 98
Fluphenazine 98
Memantine 99
Abiraterone 100
Mitoxantrone 102
Riluzole 102
Tizanidine 103
Atropine 107
Cobicistat 107
Dimethyl fumarate 108
Carbamazepine 112
Linagliptin 117
Naratriptan 117
Valproic acid 118
Fenofibric acid 120
Vecuronium 123
Vigabatrin 127
Nortriptyline 127
Asenapine 141
Desipramine 147
d
****
****
ns
% of time spent curling
0
20
40
60
80
100
L4440 => L4440
L4440 => dcr-1i
bcat-1i => bcat-1i
bcat-1i => dcr-1i
e
Mean
Replicates
0%
100%
200%
Vehicle 100
Riluzole 72
Linagliptin 75
Milnacipran 101

Supplementary Figure 4. (a) Disease indications of the 50 drugs selected for the screen, with number of drugs in parentheses. (b) Targets/ mechanisms of action of the 50 drugs, with percentage of drugs in parentheses. (c) High-throughput screen result of 50 FDA-approved drugs utilizing Coiled + Curled postures. Vehicle-treated level of *bcat-1(RNAi)* worm curling was set to 100%, and color indicates curling level relative to vehicle. Blank wells are those that had <3 worms and were excluded. (d) Post screen validation of three negative drugs, riluzole, linagliptin, and milnacipran. Vehicle-treated level of *bcat-1(RNAi)* worm curling was set to 100%, and color indicates % Coiled relative to vehicle. Blank wells are those that had <3 worms and were excluded. (e) *bcat-1* worms were moved to *dcr-1* RNAi on day 5 to disrupt RNAi machinery and curling was manually measured on day 8. n = 119 worms for L4440=>L4440, 90 worms for L4440=>*dcr-1*, 133 worms for *bcat-1*=>*bcat-1*, 110 worms for *bcat-1*=>*dcr-1*. Data are mean±s.e.m. One-way ANOVA with Tukey's post-hoc. ns, not significant. ****$p<0.0001$. Box plot shows minimum, 25th percentile, median, 75th percentile, maximum.
