# Supplementary material for: High-throughput behavioral screen in C. elegans reveals Parkinson’s disease drug candidates
Source: Commun Biol. 2021 Feb 15;4:203. doi: 10.1038/s42003-021-01731-z (PMC7884385; doi:10.1038/s42003-021-01731-z)
Supplement: Supplementary file 3 — Description of Additional Supplementary Files [file 42003_2021_1731_MOESM3_ESM.pdf]

Description of Additional Supplementary Files

File Name: Supplementary Movie 1
Description: Comparison of control and bcat-1 RNAi-fed worms showed spasm-like ‘curling’ behavior on day 8 of adulthood.

File Name: Supplementary Data 1
Description: Source Data for Fig. 2b (a), Fig. 2c (b), Fig. 2d (c), Fig. 2e (d), Fig. 3a (e), Fig. 3b (f), Fig. 3c (g), Fig. 3d (h), Fig. 3f (i), and Fig. 3g (j).
