# Supplementary material for: High-throughput behavioral screen in C. elegans reveals Parkinson’s disease drug candidates
Source: Commun Biol. 2021 Feb 15;4:203. doi: 10.1038/s42003-021-01731-z (PMC7884385; doi:10.1038/s42003-021-01731-z)
Supplement: Supplementary file 6 — Reporting Summary [file 42003_2021_1731_MOESM6_ESM.pdf]

nature research

Corresponding author(s): Dr. Coleen T. Murphy

Last updated by author(s): Dec 3, 2020

Reporting Summary

Nature Research wishes to improve the reproducibility of the work that we publish. This form provides structure for consistency and transparency in reporting. For further information on Nature Research policies, see Authors & Referees and the Editorial Policy Checklist .

Statistics

For all statistical analyses, confirm that the following items are present in the figure legend, table legend, main text, or Methods section.

n/a	Confirmed	
☐	☒	The exact sample size (*n*) for each experimental group/condition, given as a discrete number and unit of measurement
☐	☒	A statement on whether measurements were taken from distinct samples or whether the same sample was measured repeatedly
☐	☒	The statistical test(s) used AND whether they are one- or two-sided<br>*Only common tests should be described solely by name; describe more complex techniques in the Methods section.*
☐	☒	A description of all covariates tested
☐	☒	A description of any assumptions or corrections, such as tests of normality and adjustment for multiple comparisons
☐	☒	A full description of the statistical parameters including central tendency (e.g. means) or other basic estimates (e.g. regression coefficient) AND variation (e.g. standard deviation) or associated estimates of uncertainty (e.g. confidence intervals)
☒	☐	For null hypothesis testing, the test statistic (e.g. *F*, *t*, *r*) with confidence intervals, effect sizes, degrees of freedom and *P* value noted<br>*Give P values as exact values whenever suitable.*
☒	☐	For Bayesian analysis, information on the choice of priors and Markov chain Monte Carlo settings
☒	☐	For hierarchical and complex designs, identification of the appropriate level for tests and full reporting of outcomes
☒	☐	Estimates of effect sizes (e.g. Cohen's *d*, Pearson's *r*), indicating how they were calculated

Our web collection on statistics for biologists contains articles on many of the points above.

Software and code

Policy information about availability of computer code

Data collection	CeSnAP (C. elegans Snapshot Analysis Platform) is a custom code which is used to quantify C. elegans curling behavior in a high-throughput manner. The source code together with example demonstrations for CeSnAP are available on 'https://github.com/murphylab-Princeton/CeSnAP' or 'https://murphylab.princeton.edu/CeSnAP/'.
Data analysis	GraphPad Prism was used for all statistical analysis.

For manuscripts utilizing custom algorithms or software that are central to the research but not yet described in published literature, software must be made available to editors/reviewers. We strongly encourage code deposition in a community repository (e.g. GitHub). See the Nature Research guidelines for submitting code & software for further information.

Data

Policy information about availability of data

All manuscripts must include a data availability statement. This statement should provide the following information, where applicable:

- Accession codes, unique identifiers, or web links for publicly available datasets
- A list of figures that have associated raw data
- A description of any restrictions on data availability

No data with mandated deposition.

Field-specific reporting

Please select the one below that is the best fit for your research. If you are not sure, read the appropriate sections before making your selection.

Life sciences study design

All studies must disclose on these points even when the disclosure is negative.

Sample size	Exact sample size for each condition is indicated in the figure legends.
Data exclusions	Wells with fewer than 3 worms were excluded from analysis.
Replication	The drug screen was performed one time and then the top 5 drugs were further validated with software analysis of videos, revealing 4 drugs reproducibly reduced curling. One of our top drug, metformin, was further validated with manual hand counting of videos. All other experiments were repeated as indicated.
Randomization	The treatment groups were tested many times in different sequences. The order was not randomized, but was varied to account for potential covariation.
Blinding	A custom code, CeSnAP, was used for data analysis. For the most part, computer did the analysis part of this study based on a set of predefined parameters. In cases where investigators needed to override automated decision, they were blinded to the tested conditions scored on CeSnAP. Manual handcounting was not blinded.

Reporting for specific materials, systems and methods

We require information from authors about some types of materials, experimental systems and methods used in many studies. Here, indicate whether each material, system or method listed is relevant to your study. If you are not sure if a list item applies to your research, read the appropriate section before selecting a response.

Materials & experimental systems

n/a	Involved in the study	
☒	☐	Antibodies
☒	☐	Eukaryotic cell lines
☒	☐	Palaeontology
☐	☒	Animals and other organisms
☒	☐	Human research participants
☒	☐	Clinical data

Methods

n/a	Involved in the study	
☒	☐	ChIP-seq
☒	☐	Flow cytometry
☒	☐	MRI-based neuroimaging

Animals and other organisms

Policy information about studies involving animals; ARRIVE guidelines recommended for reporting animal research

Laboratory animals	The following strains of nematode worm, Caenorhabditis elegans, were used in this study: wild-type worms of the N2 Bristol strain, CF512 (fem-1(hc17); fer-15(b26)), TU3311 uIs60 (unc-119p::sid-1, unc-119p::yfp), CB113 (unc-17(e113)), TY1652 (cha-1 (y226)).
Wild animals	The study did not involve wild animals.
Field-collected samples	No field-collected samples were used.
Ethics oversight	No ethical approval or guidance was required.

Note that full information on the approval of the study protocol must also be provided in the manuscript.
